# Management of macula-on giant retinal tear detachments– outcome of pars-plana-vitrectomy with silicone oil versus gas tamponade

**DOI:** 10.1186/s12886-024-03437-2

**Published:** 2024-04-22

**Authors:** Teresa Barth, Viola Radeck, Maria-Andreea Gamulescu, Horst Helbig, David Märker

**Affiliations:** Department of Ophthalmology, University Medical Centre Regensburg, Franz-Josef-Strauß-Allee 11, 93053 Regensburg, Germany

**Keywords:** Giant retinal tear, Rhegmatogenous retinal detachment, Pars-plana-vitrectomy, Endotamponade, Silicone oil, Gas tamponade

## Abstract

**Background:**

To compare the outcome of eyes with a macula-on giant retinal tear (GRT) detachment treated with pars-plana-vitrectomy (PPV) depending on the used endotamponade.

**Methods:**

All consecutive cases with a macula-on GRT-associated rhegmatogenous retinal detachment (RRD) managed with PPV between 2007 and 2022 were retrospectively assessed depending on the selected endotamponade. By reviewing medical charts and surgical protocols the pre- and intraoperative parameters were analysed in detail. The number of vitreoretinal (VR) procedures needed for reattachment, the redetachment rate and the functional outcome were evaluated. Eyes treated with primary silicone oil (SO) tamponade were compared to eyes with primary gas tamponade. Cases with pre-existing conditions affecting outcome e.g. macula-off situation, history of trauma, status after complicated cataract surgery, former VR surgery or proliferative vitreoretinopathy grade C or higher were excluded.

**Results:**

Overall, 51 eyes of 45 patients with a macula-on GRT detachment were treated with PPV and SO (*n* = 32; 63%) or gas (*n* = 19; 37%) endotamponade in the observed period. Eyes with primary SO tamponade underwent on average 2.3 (SD 0.8) VR procedures and had a redetachment rate of 13% (*n* = 4). Eyes with gas tamponade showed a higher redetachment rate of 32% (*n* = 6) with a mean number of 1.6 (SD 1.0) PPV procedures. Postoperative best-corrected visual acuity (BCVA) was significantly better in eyes with primary gas tamponade (mean logMAR BCVA 0.32; SD 0.30) compared to eyes with SO (mean logMAR BCVA 0.60; SD 0.42; *p* = 0.008).

**Conclusions:**

Surgical management of GRT-associated RRDs is complex. In clinical routine often SO is used as endotamponade. Because of known disadvantages (second procedure necessary for SO removal, unexplained SO-related visual loss, secondary glaucoma, SO emulsification) some VR surgeons prefer a gas tamponade. In our cohort, eyes with a gas compared to SO tamponade showed higher redetachment rates. However, the final postoperative BCVA was significantly better in eyes with gas compared to SO tamponade.

**Trial registration:**

The trial protocol was approved by the local ethics committee on 25th of November 2022 (Ethikkommission der Universität Regensburg, Votum 22-3166-104).

## Background

Giant retinal tears (GRT) are defined as full-thickness breaks of three clock hours or more in circumferential extent associated with a posterior vitreous detachment (PVD) [1, 2]. Mostly GRTs arise idiopathic and affect middle-aged men [1]. Apart from spontaneous cause several risk factors like trauma, high myopia, large areas of lattice degeneration or hereditary vitreoretinopathies have been identified [2, 3]. The management of a GRT-associated rhegmatogenous retinal detachment (RRD) is challenging for vitreoretinal (VR) surgeons [4]. In macula-on situations the preoperative visual acuity can still be quite good despite the presence of an extensive tear in the outer periphery of the retina. The standard procedure for RRDs caused by a GRT is pars-plana-vitrectomy (PPV) with endotamponade [2, 5]. Because of the extent of the break, the risk of slippage of its posterior edge and the higher rate of proliferative vitreoretinopathy (PVR) silicone oil (SO) tamponade is often used [1]. SO is a well-established long-lasting endotamponade, which has been used for decades in difficult surgical situations. However, SO also has several disadvantages e.g. the need for a second PPV for its removal, the event of unexplained visual loss (UVL), the risk of secondary glaucoma and problems with SO emulsification and SO-associated keratopathy [6]. Therefore, some VR surgeons prefer a gas tamponade even in complex situations with a GRT [7]. There is no consensus whether SO or gas should be preferred in GRT detachments [3, 5, 7]. Therefore, we decided to evaluate our cases of GRT-associated RRDs, which had VR surgery at our clinic within the last fifteen years, reading their functional and anatomical results depending on the used endotamponade. To enhance comparability only cases with macula-on status were included.

## Methods

All consecutive cases, with a macula-on GRT detachment, which had VR surgery at our unit between 2007 and 2022 were retrospectively assessed. The medical records and surgical logbooks were studied in detail. The analysis comprised baseline characteristics (age, sex, site, spherical equivalent, lens status, extent and localisation of GRT), surgical parameters (cut-suture-time, laser spots, cryopexy, endotamponade) and imaging data obtained from optical coherence tomography (Spectralis®, Heidelberg Engineering). The functional outcome measured by best-corrected visual acuity (BCVA) using logarithmic decimal charts at the final follow-up and the anatomical results including redetachment rate, eventual success rate (defined as reattached retina in the absence of any endotamponade at the last follow-up visit), central foveal thickness (CFT) and presence of postoperative complications (cataract, epiretinal membrane, macular edema) were compared with regard to the primary endotamponade (SO versus gas tamponade). To define a fair endpoint postoperative BCVA values at 2 to 4 months after the last VR surgery were assessed. The following cases were included in the study: presence of a GRT ≥ three clock hours, macula-on status and at least 2 months of follow-up after the last VR surgery. Cases with a macula-off situation, PVR grade C or higher, hereditary vitreoretinopathies, history of trauma or previous VR surgery were excluded. The review of the medical records was done at least 6 months after the GRT repair.

The statistical analysis was done with SPSS statistics 28 (IBM, USA). For statistical calculation the pre- and postoperative BCVA equivalents were converted to logMAR values. Categorical variables are presented as frequency counts with percentages and continuous values as means with range and standard deviation (SD). Continuous variables were compared using student’s t-Test. For categorical variables, the x^2^ test or fisher’s exact test were used. For all statistical tests a *p*-value < 0.05 was considered significant.

## Results

Overall, 51 eyes of 45 patients underwent VR surgery between 2007 and 2002 for a macula-on GRT-associated RRD. The majority of patients were men (77%) with a mean age of 55 years (SD 9.0; range 31–73). In all eyes a PPV with endotamponade was performed. In 32 eyes (63%) SO was used. The other 19 eyes (37%) received a primary gas tamponade. Eyes with SO (SO group) were compared to eyes with gas tamponade (gas group) regarding pre-, intra- and postoperative data.

### Preoperative parameters

The baseline characteristics of cases with SO versus gas tamponade are listed in Table 1. Apart from an age difference (SO group younger than gas group), both groups were balanced with respect to preoperative parameters like sex distribution (predominantly male affected), preoperative BCVA, spherical equivalent, lens status, extent and localisation of the GRT as well as time between diagnosis and surgery. In 17 (33%) eyes a large GRT ≥ 180° was seen and in 33 (65%) cases the GRT involved the inferior 4 to 8 clock hour. Two eyes in the SO group presented with a 360° GRT.

**Table 1:** Baseline characteristics of macula-on giant retinal tear detachments before surgery

baseline characteristics	silicone oil group ( *n* = 32)	gas group (*n* = 19)	*p*-value
sex	♂ 23 (72%)	♂ 16 (84%)	0.315
age	53 (SD 9.7)	59 (SD 6.7)	0.029 #
site (right eye)	16 (50%)	8 (42%)	0.585
spherical equivalent*	-2.6 (SD 7.7)	-2.1 (SD 2.9)	0.843
lens status (phakic)	20 (63%)	12 (63%)	0.963
time diagnosis– surgery (days)	0.9 (SD 0.53; 0–2)	1.0 (SD 0.67; 0–3)	0.582
Extent of GRT (quadrants)	2 (SD 0.7; 1–4)	2 (SD 0.5; 1–3)	0.064
GRT inferiorly (4–8 o’clock)	21 (66%)	12 (63%)	0.859
GRT ≥ 180°	12 (38%)	5 (26%)	0.413
preoperative CFT (µm)	241 (SD 36.4)	228 (SD 32.7)	0.395

CFT = central foveal thickness, *eyes with pseudophakia excluded, # not significant using Bonferroni correction

### Intraoperative parameters

In all 51 eyes a PPV (2007–2010: 20-gauge, 2011–2022: 23-gauge) was performed as primary GRT repair procedure. In most phakic cases (29 of 32 phakic eyes) the PPV was combined with phacoemulsification and posterior chamber intraocular lens implantation (phakovitrectomy). In 2 eyes cataract formation was seen after the primary PPV and was treated in combination with the SO removal procedure. One eye with lens-sparing-vitrectomy and primary gas tamponade did not develop any cataract within the observation period of 11 months. Additional scleral buckling was not done. The retina was stabilised with perfluorocarbon (PFCL, F-Decalin, Fluoron GmbH, Germany) and base vitrectomy with release of traction to the edges of the GRT was completed. Cryo - and/or laserphotocoagulation was used for retinopexy at the edges of the GRT and to treat additional breaks. A 360° laser retinopexy was done in most cases (*n* = 39, 77%). After drainage of the subretinal fluid (SRF) and careful exchange of the PFCL against air tamponade, the VR surgeon selected the final type of endotamponade. In one case a direct exchange of PFCL against SO was done. SO (Oxane® 5700, Bausch + Lomb) was chosen in 32 cases. In all cases operated before 2011 with conventional 20-gauge approach SO was chosen as primary endotamponade. According to surgical logbooks the most frequent reasons for SO was the presence of a GRT itself, the presence of persistent SRF, shifting fluid, slippage of the GRT, wound-up edges of the tear or the combination of the GRT with a radial break. The other 19 eyes received a gas tamponade (EasyGas®, Fluoron GmbH, Germany: in 5% SF6, in 69% C2F6, in 26% C3F8). In Table 2 intraoperative parameters of the SO and the gas group are listed in detail. Eyes in the gas group had a significant shorter cut-suture-time (*p* < 0.001), which could be explained by the possibly more complex intraoperative situations in cases where SO was selected as tamponade and the longer duration of the air-SO exchange compared to the air-gas exchange. In addition, eyes with gas tamponade more often were treated with a 360° laser retinopexy. All other surgical parameters (laser spots, cryopexy and combination with phacoemulsification) were balanced between both groups.

**Table 2:** Intraoperative parameters of macula-on giant retinal tear detachments managed with silicone oil versus gas endotamponade

intraoperative parameters	silicone oil group ( *n* = 32)	gas group (*n* = 19)	*p*-value
cut-suture-time (minutes)	73 (SD 23.8)	50 (SD 8.5)	< 0.001 *
phakovitrectomy	18 (56%)	11 (58%)	0.909
laser spots	840 (SD 325.6)	828 (SD 250.8)	0.890
360° laser retinopexy	21 (66%)	18 (95%)	0.018 #
cryopexy	29 (91%)	17 (89%)	0.894

* significant using Bonferroni correction, # not significant using Bonferroni correction

### Functional outcome

The mean preoperative logMAR BCVA was 0.63 (SD 0.70; range 0.1–2.3). Nine patients had compromised visual acuity due to vitreous haemorrhage (VH) at presentation. The mean initial logMAR BCVA of all eyes without VH was 0.31 (SD 0.28; range 0.1–1.5). There was no statistical significant difference in preoperative BCVA values between the two groups (*p* = 0.282). The mean postoperative BCVA of all cases was 0.49 (SD 0.40) at the endpoint, which was on average assessed 2.5 months (SD 0.707; range 2–4) after the last VR procedure. At the endpoint eyes after gas tamponade (mean BCVA 0.32; SD 0.30) had a statistically significant better BCVA than eyes after SO (mean BCVA 0.60; SD 0.42) tamponade (*p* = 0.008). Table 3 lists all BCVA values of eyes with macula-on giant GRT detachments before and after PPV.

**Table 3:** Functional development of macula-on giant retinal tear detachments before and after PPV

BCVA and IOP	silicone oil group ( *n* = 32)	gas group (*n* = 19)	*p*-value
preoperative logMAR BCVA	0.70 (SD 0.75)	0.48 (SD 0.58)	0.282
preoperative logMAR BCVA #	0.31 (SD 0.30)	0.31 (SD 0.24)	0.969
postoperative logMAR BCVA	0.60 (SD 0.42)	0.32 (SD 0.30)	0.008*
postoperative logMAR BCVA ≤ 0.3	14 (44%)	13 (68%)	0.073
Difference BCVA (lines) #	-3 (SD 0.9)	+ 1 (SD 1.2)	0.017
IOP min (mmHg)	12 (SD 3.1)	11 (SD 3.0)	0.192
IOP max (mmHg)	22.7 (SD 9.6)	21.5 (8.8)	0.727

BCVA = best-corrected visual acuity, IOP = intraocular pressure, PPV = pars-plana-vitrectomy, # eyes with vitreous haemorrhage at initial presentation excluded, * significant using Bonferroni correction

### Postoperative parameters

A primary surgical success (defined as reattached retina after two VR procedures in SO group including PPV and the SO removal procedure or a successful reattachment after one PPV and resolution of the endotamponade in the gas group) was seen in 41 (80%) eyes. A redetachment happened in 4 (13%) cases of the SO group and in 6 (32%) eyes of the gas group. In 6 of 10 cases the redetachment involved the macula. The reasons for failure were the development of secondary PVR (*n* = 5), an elevated GRT edge (*n* = 2) or retinal slippage (*n* = 1). Final reattachment of the retina was achieved in all eyes. In one case the patient refused the SO removal. In all other eyes with primary SO tamponade the SO was removed after a mean interval of 19 weeks (SD 17.5). Overall, eyes with a primary gas tamponade needed fewer VR procedures for eventual success than eyes in the SO group. When comparing the number of PPVs for final reattachment without counting the SO removal procedure, there was no difference between the two groups (*p* = 0.192). Eyes in the gas group had significantly fewer postoperative complications compared to the eyes with SO (*p* = 0.007). The incidence of UVL, defined as loss of ≥ 3 Snellen lines without any apparent reason, occurred in 50% of the eyes with primary SO tamponade and in no eye in the gas group. In eyes with UVL the SO was removed later than in eyes without UVL, but the difference was statistically not significant [124 days (SD 57.9) of SO tamponade in eyes with UVL compared to 99 days (SD 53.4;) of SO filling in eyes without UVL ( *p* = 0.241)]. The shortest duration of SO tamponade in an eye, that experienced UVL, was 73 days. When comparing the incidence of the other postoperative complications (macular edema, epiretinal membranes, IOP elevation) no statistical significant difference was found between the SO and the gas group (*p* = 0.682). Details of the postoperative parameters are listed in Table 4. There was no difference in CFT between the SO and the gas group at the final visit (*p* = 0.928). The final follow-up visit was done on average 14 months (SD 22.7; range 2-129) after the last VR procedure. In 23 (45%) of the cases the fellow eye also developed a RRD, which was related to a GRT in 8 (16%) eyes. Fellow eye prophylaxis with 360° laser- or cryopexy was not done. The mean interval between the first and second eye involvement was 27 months (SD 32.4; range 2-104).

**Table 4:** Surgical outcome of macula-on giant retinal tear detachments after silicone oil versus gas endotamponade

surgical outcome	silicone oil group (*n* = 32)	gas group (*n* = 19)	*p*-value
overall number of PPVs	2.3 (SD 0.8)	1.6 (SD 1.0)	0.020 #
redetachment rate	4 (13%)	6 (32%)	0.099
eyes with postoperative complications	24 (75%)	7 (37%)	0.007 *
eyes with postoperative complications without UVL	10 (26%)	7 (37%)	0.682
list of postoperative complications			
- UVL - IOP increase > 30 mmHg - epiretinal membrane - macular edema	12 (50%) 1 (3%) 4 (13%) 9 (28%)	0 (0%) 1 (5%) 3 (16%) 3 (16%)	
postoperative CFT (µm)	261 (SD 41.9)	262 (SD 47.7)	0.928
follow-up after final surgery (months)	17 (SD 26.6)	10 (SD 14.3)	
fellow eye detachment	12 (38%)	11 (58%)	

BCVA = best-corrected visual acuity, PPV = pars-plana-vitrectomy, UVL = unexplained visual loss, CFT = central foveal thickness, * significant using Bonferroni correction, # not significant using Bonferroni correction

## Discussion

GRT-associated detachments are challenging because of the great mobility of the retina, the possible complication of slippage of the posterior edge of the tear with fold formation and the higher risk of PVR [2]. There are different opinions about the best surgical approach for GRT fixation. Schepens was the first to describe the entity of GRTs and recommended surgical management depending on GRT size and mobility, lens status and presence of PVR. For GRTs smaller than two quadrants without slippage a 360° scleral buckle and cryopexy with or without internal gas tamponade was done in earlier days and resulted in a success rate of 50–61% [2]. However, a GRT of more than 180° had a success rate of only 11–14% [2, 3]. With the introduction of PPV in the 1970ies the initial success rate raised to about 86% with a gas tamponade, but required intraoperative manoeuvres like rotation of the operating table and kneeling of the surgeon on the floor below the patient during gas injection [8]. Still, the secondary development of PVR was an issue and resulted in a redetachment rate of up to 65% [3, 8]. With the use of SO as endotamponade success rates of up to 93% were reported in the 1990ies [3]. Recently novel surgical techniques, the introduction of small-gauge-vitrectomy and the use of PFCL has considerably improved the outcome [9, 10].

Nowadays, a GRT-associated detachment is mostly treated with PPV, PFCL and endotamponade optionally combined with a 360° scleral buckle [11]. In cases with limited GRT extent lens-sparing vitrectomy is possible (one patient with a three clock hour tear in our cohort). In most cases the PPV is also combined with a lens extraction to attain good peripheral visualization and better accessibility of the vitreous base [4]. The need for an encircling band is controversial [12]. While Goezinne et al. reported lower redetachment rates due to encircling band placement in GRTs, other authors found no improvement in recurrence rates with the combination of PPV and a buckle [11, 13, 14]. In our cohort, additional scleral buckling was not done, which alleviates comparison of the SO and the gas group.

In many centres around the world SO is preferred as primary tamponade [10, 11, 15]. SO has some advantages like a long-lasting tamponade effect, lower risk of secondary PVR, less slippage and immediate postoperative visualization of the retina and better vision for the patient in the early postoperative phase [6]. But, SO also has relevant disadvantages like the need for a SO removal procedure, corneal changes, intraocular pressure (IOP) elevation, SO emulsification, macular edema and UVL [6, 16].. In our series, eyes after primary SO tamponade had a statistically significant worse visual outcome than after gas tamponade, which is in concordance with other reports [5, 7]. Especially considering the high incidence of UVL in fovea-sparing GRTs treated with SO, Banerjee et al. support the idea of a “move away from silicone oil” towards long-lasting gas especially in eyes with a macula-on situation [5]. Other reports did not find any significant difference in final BCVA or rate of complications after GRT repair with SO compared to gas tamponade, but in these studies also macula-off detachments were included [9, 17]. In our series, the eyes after SO tamponade had significantly more postoperative complications, this being mainly generated by the high percentage of UVL (50%). Some authors discuss, whether the duration of SO may be a risk factor for UVL [18, 19]. In our series, the mean duration of SO tamponade was similar to other reports ranging between 3 and 5 months [5, 18]. According to the literature, the minimum duration of SO tamponade in eyes, which experienced UVL, was about 3 months [5, 18]. Our cases with UVL had in general a longer SO tamponade duration (124 days) than eyes without UVL (99 days), but the difference was statistically not significant. Maybe, the quicker removal of SO (within 3 months) could be an option in GRT cases with a macula-on status to reduce the risk of UVL. However, the data availability here is poor to identify the ideal SO duration to prevent UVL and to not risk a higher redetachment rate.

Considering redetachment rate, the overall redetachment rate after GRT repair was 20%, which is similar to other reports [11, 14, 20]. In agreement with other authors, eyes with a primary SO tamponade had a lower redetachment rate compared to eyes with gas [20]. The main reason for recurrent RRD in our study was the development of PVR, which is in concordance with other reports [11]. The use of SO is known to lower the risk of PVR, which could explain the lower redetachment rates in eyes with SO tamponade. In a retrospective series by Li e al. from a centre in the US a comparable redetachment rate of 31% for GRT repair with a primary gas tamponade was found [21]. Interestingly, Li et al. also included eyes with complex GRTs after trauma or with PVR grade C and achieved a single surgery success rate of 75%, while most cases (81%) were treated with PPV, PFCL and gas. However, functional outcome was not reported separately for eyes with gas versus eyes with SO in this study [21].

Several studies reporting the outcome of GRT management have been published [1, 4, 7]. But alongside with Banerjee et al. [5], our study is the only one, which compares solely the outcome of GRT-associated detachments with a macula-on status. We acknowledge the limitations of our study, which are restricted by its monocentric character, the retrospective design, the inclusion of both eyes in patients with bilateral GRT and the case-specific selection of the endotamponade, which is strongly biased by the intraoperative situation and the experience of the surgeon. A prospective randomized clinical trial would be desirable, but remains difficult due to the rarity of GRTs. The strengths of our study are the balanced baseline characteristics like preoperative BCVA, GRT extent and localisation, which were homogenously distributed between the gas and the SO group.

## Conclusions

Overall, there are good arguments for a SO tamponade for GRT repair like prophylaxis of retinal slippage and lower risk of PVR and redetachment [3]. On the other hand, eyes with a gas tamponade benefit from a better functional outcome and less VR procedures. In view of our own results and the data from several other authors [5, 7, 21], a primary gas tamponade in GRT repair is an option which should be considered, especially in patients with a macula-on situation. Alternatively, a faster removal of the SO tamponade would be an option to prevent the risk of visual loss. In the end, the endotamponade is selected depending on the surgeon’s choice and the intraoperative situation and, with the words of Schepens, “tailored to the specific clinical findings in each case” [2].

## Data Availability

The data that support the findings of this study are available from teresa.barth@ukr.de upon reasonable request and with permission of the local ethics committee.

## Abbreviations

BCVA: best corrected visual acuity
CFT: central foveal thickness
GRT: giant retinal tear
IOP: intraocular pressure
OCT: optical coherence tomography
PFCL: perfluorocarbon liquids
PVD: posterior vitreous detachment
PVR: proliferative vitreoretinopathy
RRD: rhegmatogenous retinal detachment
SO: silicone oil
UVL: unexplained vision loss
VR: vitreoretinal
